# Thyroid autoimmunity is associated with increased uterine artery blood flow resistance and predicts adverse pregnancy outcomes in women with unexplained recurrent pregnancy loss: a retrospective cohort study

**DOI:** 10.3389/fendo.2026.1928429

**Published:** 2026-09-07

**Authors:** Ruifang Wang, Lili Xiong, Mengmeng Jia, Shuangran Chen, Wenjuan Mei

**Affiliations:** 1 Department of Obstetrics and Gynecology, The First Affiliated Hospital, and College of Clinical Medicine of Henan University of Science and Technology, Luoyang, China; 2 Department of Medical Ultrasonic, The First Affiliated Hospital, and College of Clinical Medicine of Henan University of Science and Technology, Luoyang, China

**Keywords:** pregnancy outcome, subsequent pregnancy loss, thyroid autoimmunity, unexplained recurrent pregnancy loss, uterine artery blood flow parameters

## Abstract

**Background:**

Uterine artery blood flow resistance is critical for endometrial receptivity and successful implantation. Thyroid autoimmunity (TAI) is prevalent in recurrent pregnancy loss (RPL), but whether TAI affects uterine hemodynamics before conception and whether this predicts subsequent pregnancy outcomes has not been directly investigated.

**Objective:**

This study aimed to determine whether TAI is associated with increased uterine artery resistance in non-pregnant women with unexplained RPL (URPL) and whether elevated resistance indices predict adverse subsequent pregnancy outcomes, thereby providing evidence for preconception risk stratification.

**Methods:**

A total of 486 non-pregnant women with URPL were enrolled in this retrospective cohort study. Serum TPOAb, TGAb, TSH, FT4 and FT3 were measured. TAI and non-TAI, based on their thyroid antibody status. TAI was defined as TPOAb ≥60IU/mL and/or TGAb ≥60 IU/mL. All underwent transvaginal Doppler ultrasound to measure bilateral uterine artery resistance index (RI), pulsatility index (PI), and peak systolic velocity/end diastolic velocity (S/D). We assessed the incidence of pregnancy, subsequent pregnancy loss, and live birth. Logistic regression analysis was performed to examine the associations between TAI, uterine artery blood flow parameters and subsequent pregnancy loss.

**Results:**

TAI women (n=242) had significantly higher RI, PI and S/D values compared with non-TAI women (n=244) (left RI: 0.82[0.77, 0.85] vs. 0.77 [0.73, 0.83], P <0.001; right RI: 0.82[0.77, 0.85] vs. 0.78 [0.73, 0.84], P <0.001; Right PI: 2.36[2.19, 2.59] vs. 2.29[1.89, 2.56], P = 0.010; Left S/D: 5.56[4.00, 6.25] vs. 4.35[3.70, 5.88], P <0.001; Right S/D: 5.56[4.35, 6.67] vs. 4.55[3.70, 6.25], P <0.001). During follow-up, 240 of 486 women achieved a next pregnancy (49.3%). The clinical pregnancy rate did not differ significantly between the TAI and non-TAI groups (42.6% vs. 48.0%, P = 0.379). Among those who conceived, TAI women had a significantly higher rate of recurrent pregnancy loss (28.7% vs. 16.0%) and a lower live birth rate (71.3% vs. 84.0%) (P = 0.018). Logistic regression showed that TAI positivity (aOR=2.02, 95%CI 1.08-3.87), mean RI (aOR=1.96, 95%CI 1.3-3.00), mean PI (aOR=2.24, 95%CI 1.58-3.30), and mean S/D (aOR=3.567, 95%CI 1.03-1.90) were independent predictors of subsequent pregnancy loss.

**Conclusion:**

Thyroid autoimmunity is associated with increased uterine artery blood flow resistance in non-pregnant URPL women. TAI positivity and elevated uterine artery Doppler indices (mean RI, PI and S/D) were independently associated with subsequent pregnancy loss.

## 1 Introduction

Recurrent pregnancy loss (RPL) affects approximately 1-3% of women of childbearing age and is defined as two or more consecutive spontaneous losses before 12 weeks of gestation without an identified cause after routine evaluation (1, 2). Despite extensive testing, nearly 50% of RPL cases remain unexplained, defined as unexplained RPL(URPL), placing a heavy psychological and physical burden on affected couples (3, 4).

Uterine artery blood flow is a key determinant of endometrial receptivity and successful embryo implantation. Increased resistance in the uterine arteries, reflected by elevated resistance index (RI) and pulsatility index (PI), has been observed in women with RPL during the mid-luteal phase (5, 6). However, the underlying factors that cause high uterine artery resistance in URPL patients are not fully understood.

Thyroid autoimmunity (TAI), defined as the presence of thyroid peroxidase antibodies (TPOAb) and/or thyroglobulin antibodies (TGAb) with euthyroid function, is one of the most common autoimmune conditions in women of reproductive age (7). TAI is more prevalent in RPL populations compared with fertile controls (8, 9). Emerging evidence suggests that TAI is associated with adverse pregnancy outcomes even in euthyroid women, but the mechanisms remain elusive (10–12).

While our prior work established the prognostic value of TAI, the present study adds a novel dimension by integrating uterine artery Doppler parameters into the risk assessment model. Additionally, as this cohort is drawn from a separate institution with no patient overlap, it simultaneously provides an independent validation of the TAI-outcome association. Recently, placental studies have shown that TAI-positive women have a higher risk of maternal vascular malperfusion, suggesting impaired uterine-placental circulation (13). However, whether TAI directly increases uterine artery blood flow resistance in the non-pregnant state has not been investigated. Therefore, the present study aimed to explore the association between TAI and uterine artery Doppler parameters in non-pregnant women with URPL.

## 2 Materials and methods

### 2.1 Study population

This retrospective cohort study included non-pregnant women with a history of URPL who underwent mid-luteal uterine artery Doppler assessment and thyroid antibody measurement as part of routine pre-conception evaluation between June 2024 and October 2025 at the Department of Obstetrics and Gynecology of the First Affiliated Hospital, and College of Clinical Medicine of Henan University of Science and Technology. URPL was defined as ≥ 2 spontaneous pregnancy losses before 12 weeks of gestation after excluding known causes (parental chromosomal abnormalities, uterine anomalies, antiphospholipid syndrome, other autoimmune diseases, and abnormal thyroid function). Exclusion criteria included: use of anticoagulants, immunomodulators, or hormones within the previous 3 months; TSH, FT3 or FT4 outside the normal range; known diabetes or hypertension; pregnancy through assisted reproductive conception; with BMI (body mass index) ≥ 28kg/m2 (the Chinese obesity threshold), and current pregnancy. Participants were selected in a frequency-balanced manner such that comparable numbers of TAI-positive and TAI-negative participants were accrued.

This study was approved by the Ethics Committee of the First Affiliated Hospital, and College of Clinical Medicine of Henan University of Science and Technology (Approval No. 2024-0684). The procedures used in this study adhere to the tenets of the Declaration of Helsinki.

### 2.2 Laboratory measurements

Laboratory data were extracted from electronic medical records. Peripheral venous blood samples were collected after an overnight fast and processed within 2 hours. Serum TPOAb, TGAb, FT4, FT3, and TSH were measured by electrochemiluminescence immunoassay on a Roche Cobas e801 analyzer (Roche Diagnostics, Mannheim, Germany). The analytical sensitivity was 5.0 IU/mL for TPOAb and 10.0 IU/mL for TGAb; intra-assay and inter-assay coefficients of variation were <5% and <8%, respectively, for all assays. fasting blood glucose (FBG), cholesterol (CHO), and triglycerides (TG) were measured on a Roche Cobas c702 analyzer. TAI was defined as TPOAb ≥ 60 IU/mL and/or TGAb ≥ 60 IU/mL in the presence of euthyroid function, using the manufacturer’s stated reference limits (TPOAb: <60 IU/mL; TGAb: <60 IU/mL). Euthyroid status was defined as TSH (0.27-4.40 μIU/mL), FT4 (0.93-1.70 ng/dL), and FT3 (2.0-4.4 pg/mL) within the reference ranges.Non-TAI was defined as TPOAb < 60 IU/mL and TGAb <60 IU/mL with euthyroid function.

### 2.3 Uterine artery Doppler ultrasonography

Transvaginal color Doppler ultrasonography was performed during the mid-luteal phase (cycle days 19-23) by a single examiner with 8 years of experience in gynecological Doppler, using a Voluson E8 system (GE Healthcare Austria GmbH & Co OG) with a 5–9 MHz transvaginal transducer. The examiner was blinded to the participants’ thyroid antibody results. The uterine artery was identified with color Doppler at the level of the internal cervical os, and the ascending branch was interrogated with an insonation angle below 30 degrees. The sample volume was set at 2 mm, wall filter at 50 Hz. Once three consecutive uniform waveforms were obtained, RI, PI, and S/D were calculated automatically by the system’s software, and the mean of three cardiac cycles was recorded. Intra-observer reproducibility was assessed in 30 randomly selected participants examined twice within the same session; the intraclass correlation coefficient was 0.94 (95% CI: 0.89-0.97) for RI and 0.91 (95% CI: 0.85-0.95) for PI.

### 2.4 Follow-up for subsequent pregnancy outcomes

All enrolled women were advised to attempt pregnancy naturally. Clinical outcome data were extracted from electronic medical records. Participants were followed through medical record review until clinical pregnancy, until 12 months had elapsed without conception, or until the follow-up census date of June 30, 2026, whichever occurred first. Clinical pregnancy was defined as the ultrasonographic demonstration of an intrauterine gestational sac with fetal cardiac activity. Biochemical pregnancies (a positive urine or serum β-hCG result without subsequent ultrasound confirmation) were recorded separately and are reported in **Supplementary Table 3**; they were not counted as clinical pregnancies. The following outcomes were recorded as follows: Clinical pregnancy rate: the proportion of women achieving an intrauterine gestational sac with a fetal heartbeat. Recurrent pregnancy loss (RPL) rate: the proportion of pregnant women experiencing another pregnancy loss before 24 weeks of gestation (including biochemical pregnancy). Live birth (LB) rate: the proportion of pregnant women delivering a live infant beyond 24 weeks of gestation. Woman who did not conceive within 12 months were recorded as non-pregnant.

### 2.5 Statistical analysis

The sample size was determined by the number of eligible women enrolled during the study period. With 486 participants, the study had 80% power to detect a between-group difference in RI of 0.02 (assuming SD 0.07, α = 0.05).Continuous variables were expressed as mean ± standard deviation. Non-normally distributed continuous variables were expressed as median (P25, P75) and compared using the Mann–Whitney U test. Categorical variables were compared using the chi-square test. Spearman correlation analysis was used to evaluate the relationship between antibody titers and Doppler parameters, and the correlation analysis is exploratory and that P values were not unadjusted. In addition to previous methods, differences in pregnancy outcomes between TAI and non-TAI groups were compared using chi-square test. Logistic regression was used to identify independent predictors of subsequent pregnancy loss, including TAI status and uterine artery blood flow parameters. Firth penalized logistic regression was used to build predictive models, addressing sparse-data bias. After normality testing, t-tests or Wilcoxon rank-sum tests were used for univariate screening (p<0.1), followed by AIC-based stepwise regression for final variable selection, with forced adjustment for age, BMI, number of prior pregnancies, and TSH. Model discrimination was assessed by AUC, calibration by calibration plots and Brier score. Internal validation employed 200 bootstrap resamples to obtain bias-corrected performance estimates with 95% confidence intervals. All analyses were performed using SPSS version 27.0 and R software (R4.4.3).

## 3 Results

### 3.1 Baseline characteristics of the study population

A total of 486 women with URPL were enrolled, of whom 242 (49.8%) were TAI and 244 (50.2%) were non-TAI. The near-equal group distribution reflects frequency-matched selection to ensure adequate statistical power for between-group comparisons, rather than consecutive sampling; therefore, these proportions do not estimate the population prevalence of TAI in URPL. In TAI group, there were 164(67.8%) women with TPOAb-positive only, 37(15.3%) women with TGAb-positive only, and 41(16.9%) women with positive for both. Baseline characteristics are shown in **Table 1**. No significant differences were observed between the two groups in age, BMI, TSH, FT4, FT3, FBG, CHO, or TG (all P > 0.05) (**Table 1**).

**Table 1:** Baseline characteristics of the study population.

Parameter	TAI (n=242)	Non-TAI (n=244)	*P* value
Age (years)	32.00 (30.00, 35.00)	31.00 (28.25, 35.00)	0.122
BMI (kg/m²)	22.90 (22.00, 24.40)	23.10 (21.70, 24.40)	0.238
Number of prior PL	3.00 (3.00, 4.00)	3.00 (3.00, 4.00)	**0.025**
TSH (μIU/mL)	1.79 (1.34, 2.46)	1.85 (1.32, 2.36)	0.525
FT4 (ng/dL)	1.26 (1.18, 1.41)	1.26 (1.12, 1.44)	0.416
FT3 (pg/mL)	2.96 (2.53, 3.31)	2.91 (2.49, 3.31)	0.650
Glucose (mmol/L)	4.71 (4.37, 4.99)	4.74 (4.37, 5.05)	0.586
Cholesterol (mmol/L)	3.60 (3.10, 4.14)	3.67 (3.10, 4.22)	0.318
Triglycerides (mmol/L)	1.33 (0.96, 1.62)	1.29 (0.93, 1.61)	**0.029**
TPOAb (IU/mL)	66.25(62.38, 74.48)	21.95 (18.50, 24.78)	**<0.001**
TGAb (IU/mL)	54.15 (46.08, 61.03)	18.41 (15.13, 20.27)	**<0.001**

Non-normally distributed continuous variables were expressed as median (P25, P75) and compared using the Mann–Whitney U test. *P* values <0.05 were shown in bold. TAI, thyroid autoimmunity; BMI, body mass index; PL, pregnancy loss; TSH, thyroid-stimulating hormone; FT4, free thyroxine; FT3, free triiodothyronine; RI, resistance index; PI, pulsatility index; S/D, systolic/diastolic ratio; TPOAb, thyroid peroxidase antibody; TGAb, thyroglobulin antibody.

### 3.2 Comparison of uterine artery Doppler parameters

**Figure 1:**
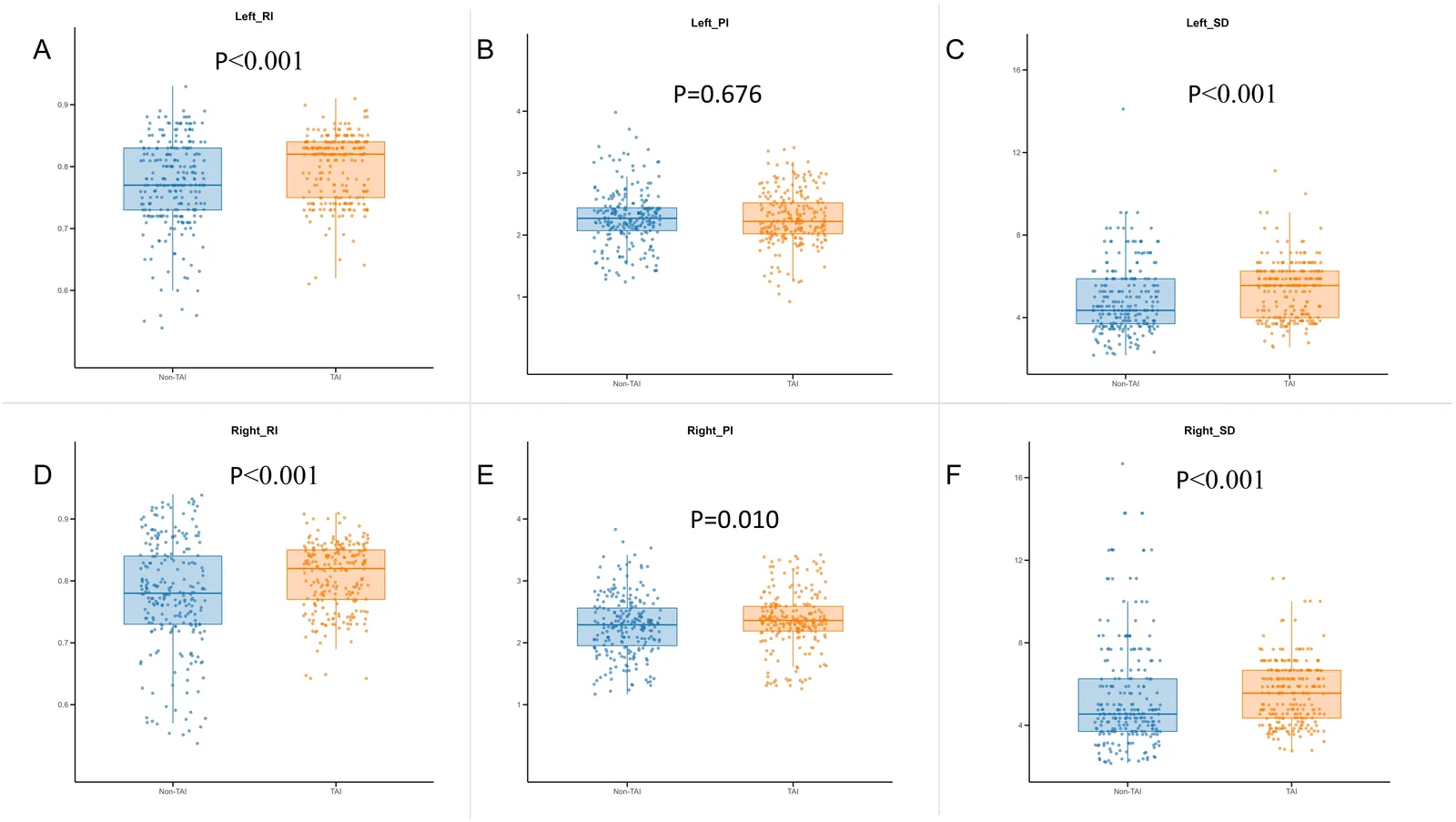
Comparison of RI, PI and S/D levels between TAI and non-TAI groups. **(A–C)** Left RI, PI and S/D. **(D–F)** Right RI, PI and S/D. Box plots show the median and interquartile range with individual data points. p values were derived from the Mann–Whitney U test.

As shown in **Figure 1** and **Table 2**, TAI group had significantly higher baseline bilateral RI, right uterine artery PI and bilateral S/D with non-TAI group [left RI: 0.82(0.77, 0.85) vs. 0.77 (0.73, 0.83), P <0.001; right RI: 0.82(0.77, 0.85) vs. 0.78 (0.73, 0.84), P <0.001; Right PI: 2.36(2.19, 2.59) vs. 2.29(1.89, 2.56), P = 0.010; Left S/D: 5.56(4.00, 6.25) vs. 4.35(3.70, 5.88), P <0.001; Right S/D: 5.56(4.35, 6.67) vs. 4.55(3.70, 6.25), P <0.001]. Only left PI did not differ significantly between the two groups (P > 0.05).

**Table 2:** Uterine artery Doppler parameters in TAI and non-TAI groups.

Parameter	TAI (n=242)	Non-TAI (n=244)	*P* value
Left uterine artery RI	0.82(0.77, 0.85)	0.77(0.73, 0.83)	**<0.001**
Right uterine artery RI	0.82(0.77, 0.85)	0.78(0.73, 0.84)	**<0.001**
Left uterine artery PI	2.22(2.02, 2.53)	2.27(2.07, 244)	0.676
Right uterine artery PI	2.36(2.19, 2.59)	2.29(1.894, 2.56)	**0.010**
Left uterine artery S/D	5.56(4.00, 6.25)	4.35(3.70, 5.88)	**<0.001**
Right uterine artery S/D	5.56(4.35, 6.67)	4.55(3.70, 6.25)	**<0.001**

Non-normally distributed continuous variables were expressed as median (P25, P75) and compared using the Mann–Whitney U test. *P* values <0.05 were shown in bold. RI, resistance index; PI, pulsatility index; S/D, systolic/diastolic ratio.

### 3.3 Correlation analysis

Spearman’s correlation analysis revealed that TPOAb titers were positively correlated with bilateral uterine artery RI (left RI: r = 0.209, P <0.001; Right RI: r = 0.173, P <0.001), right uterine artery PI (r = 0.110, P = 0.015, and bilateral uterine artery S/D (left S/D: r = 0.208, P <0.001; Right S/D: r = 0.174, P <0.001). TGAb titers showed a positive correlation with bilateral uterine artery RI (left RI: r = 0.265, P <0.001; Right RI: r = 0.149, P <0.001), right uterine artery PI (r = 0.103, P = 0.024, and bilateral uterine artery S/D (left S/D: r = 0.264, P <0.001; Right S/D: r = 0.150, P <0.001). Furthermore, TAI status has significant correlations with bilateral uterine artery RI (left RI: r = 0.209, P <0.001; Right RI: r = 0.160, P <0.001), right uterine artery PI (r = 0.117, P = 0.010, and bilateral uterine artery S/D (left S/D: r = 0.208, P <0.001; Right S/D: r = 0.161, P <0.001) (**Table 3**).

**Table 3:** Correlation between RI, PI, S/D levels and TPOAb titers, TGAb titers, TAI status in the study population.

Parameter	Left-RI	Left -PI	Left-S/D	Right-RI	Right-PI	Right-S/D
TPOAb (IU/ml)						
r_1_	**0.209** **(0.119-0.295)**	**-0.018** **(-0.109-0.074)**	**0.208** **(0.119-0.294)**	**0.173** **(0.083-0.261)**	**0.110** **(0.019-0.200)**	**0.174** **(0.084-0.261)**
*P_1_*	**<0.001**	**0.690**	**<0.001**	**<0.001**	**0.015**	**<0.001**
TGAb (IU/ml)						
r_2_	**0.265** **(0.178-0.348)**	**-0.012** **(-0.104-0.079)**	**0.264** **(0.177-0.348)**	**0.149** **(0.059-0.238)**	**0.103** **(0.011-0.192)**	**0.150** **(0.059-0.238)**
*P_2_*	**<0.001**	**0.789**	**<0.001**	**<0.001**	**0.024**	**<0.001**
TAI status						
r_3_	**0.209** **(0.119-0.295)**	**-0.019** **(-0.110-0.073)**	**0.208** **(0.119-0.294)**	**0.160** **(0.070-0.248)**	**0.117** **(0.026-0.206)**	**0.161** **(0.070-0.249)**
*P_3_*	**<0.001**	**0.677**	**<0.001**	**<0.001**	**0.010**	**<0.001**

Data were rho(95%CI). *P* values <0.05 were shown in bold. Spearman’s rho and coefficients were used to assess the correlation between TPOAb titers, TGAb titers, TAI status and RI, PI, S/D levels. _1_ Spearman’s correlation analysis between TPOAb titers and RI, PI, S/D levels; _2_ Spearman’s correlation analysis between TGAb titers and RI, PI, S/D levels; _3_ Spearman’s correlation analysis between TAI status and RI, PI, S/D levels. RI, resistance index; PI, pulsatility index; S/D, systolic/diastolic ratio.

### 3.4 Follow-up and subsequent pregnancy outcomes

**Figure 2:**
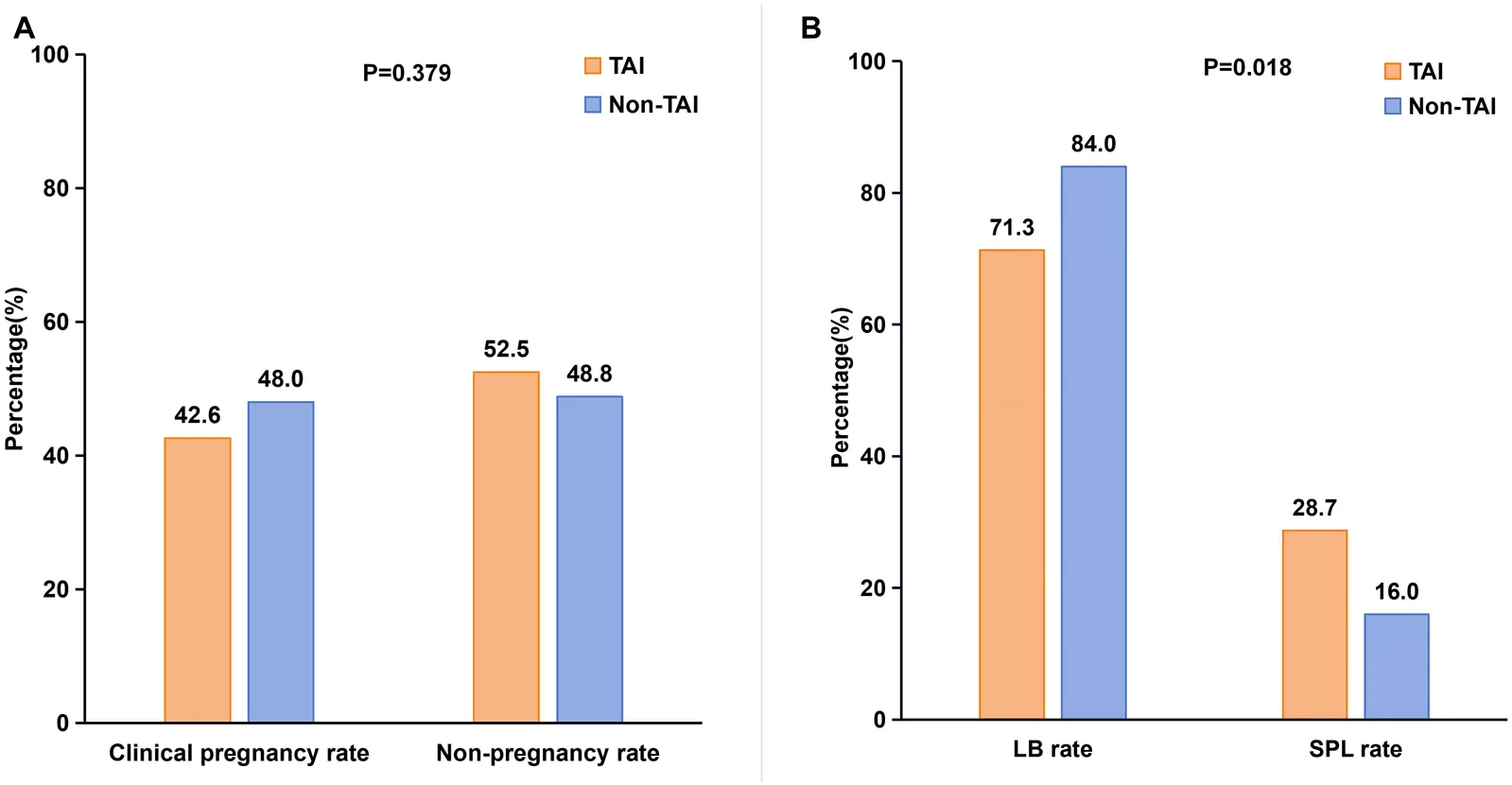
Comparison of pregnancy outcomes between TAI group and non-TAI group (%). **(A)** Comparison of pregnancy rates. **(B)** Comparison of subsequent pregnancy outcomes. LB, live birth; SPL, subsequent pregnancy loss.

Among the 486 women, 240 (49.3%) achieved a clinical pregnancy within 12 months of follow-up. The clinical pregnancy rate did not differ significantly between the TAI and non-TAI groups (42.6% vs. 48.0%, P = 0.379) (**Table 4**; **Figure 2**).

**Table 4:** Pregnancy outcomes according to TAI status.

Outcomes	TAI (n=242)	Non-TAI (n=244)	*P* value
Non-pregnancy rate	127 (52.5%)	119 (48.8%)	**0.044**
Subsequent pregnancy loss rate	33 (13.6%)	20 (8.2%)	
Live birth rate	82 (33.9%)	105 (43.0%)	

Values are n/N (%). P values <0.05 were shown in bold.

Among the 240 women who conceived, 53 (22.1%) experienced a subsequent pregnancy loss, and 187 (77.9%) had a live birth. The subsequent pregnancy loss rate was significantly higher in the TAI-positive group (28.7% vs. 16.0%, P = 0.018), and the live birth rate was significantly lower (71.3% vs. 84.0%) (**Table 4**; **Figure 2**).

### 3.5 Association between uterine artery Doppler parameters and pregnancy outcomes

To further explore whether the increased uterine artery resistance in TAI women contributes to adverse outcomes, we compared Doppler parameters between women who subsequently had a live birth versus those who experienced another pregnancy loss. As shown in **Table 5**, women with subsequent pregnancy loss had significantly higher left RI, right PI and left S/D values at baseline than those who had a live birth (left RI: 0.83[0.78, 0.84] vs. 0.78 [0.73, 0.83], P <0.001; Right PI: 2.48[2.27, 2.85] vs. 2.16[1.83, 2.37], P <0.001; Left S/D: 5.87[4.55, 6.25] vs. 4.55[3.70, 5.88], P <0.001). And right RI, right PI and right S/D did not differ significantly between the two outcome groups (all P values> 0.05).

**Table 5:** Baseline uterine artery Doppler parameters by subsequent pregnancy outcome.

Parameter	Subsequent live birth (n=187)	Subsequent pregnancy loss (n=53)	*P* value
Left uterine artery RI	0.78 (0.73, 0.83)	0.83 (0.78, 0.84)	**<0.001**
Right uterine artery RI	0.78 (0.73, 0.84)	0.80 (0.76, 0.86)	0.050
Left uterine artery PI	2.15 (1.88, 2.39)	2.19 (1.93, 2.38)	0.598
Right uterine artery PI	2.16 (1.83, 2.37)	2.48 (2.27, 2.85)	**<0.001**
Left uterine artery S/D	4.55 (3.70, 5.88)	5.87 (4.55, 6.25)	**<0.001**
Right uterine artery S/D	4.55 (3.70, 6.25)	5.00 (4.17, 7.14)	0.051

Non-normally distributed continuous variables were expressed as median (P25, P75) and compared using the Mann–Whitney U test. *P* values <0.05 were shown in bold. RI, resistance index; PI, pulsatility index; S/D, systolic/diastolic ratio.

### 3.6 Logistic regression analysis for predictors of subsequent pregnancy loss

**Figure 3:**
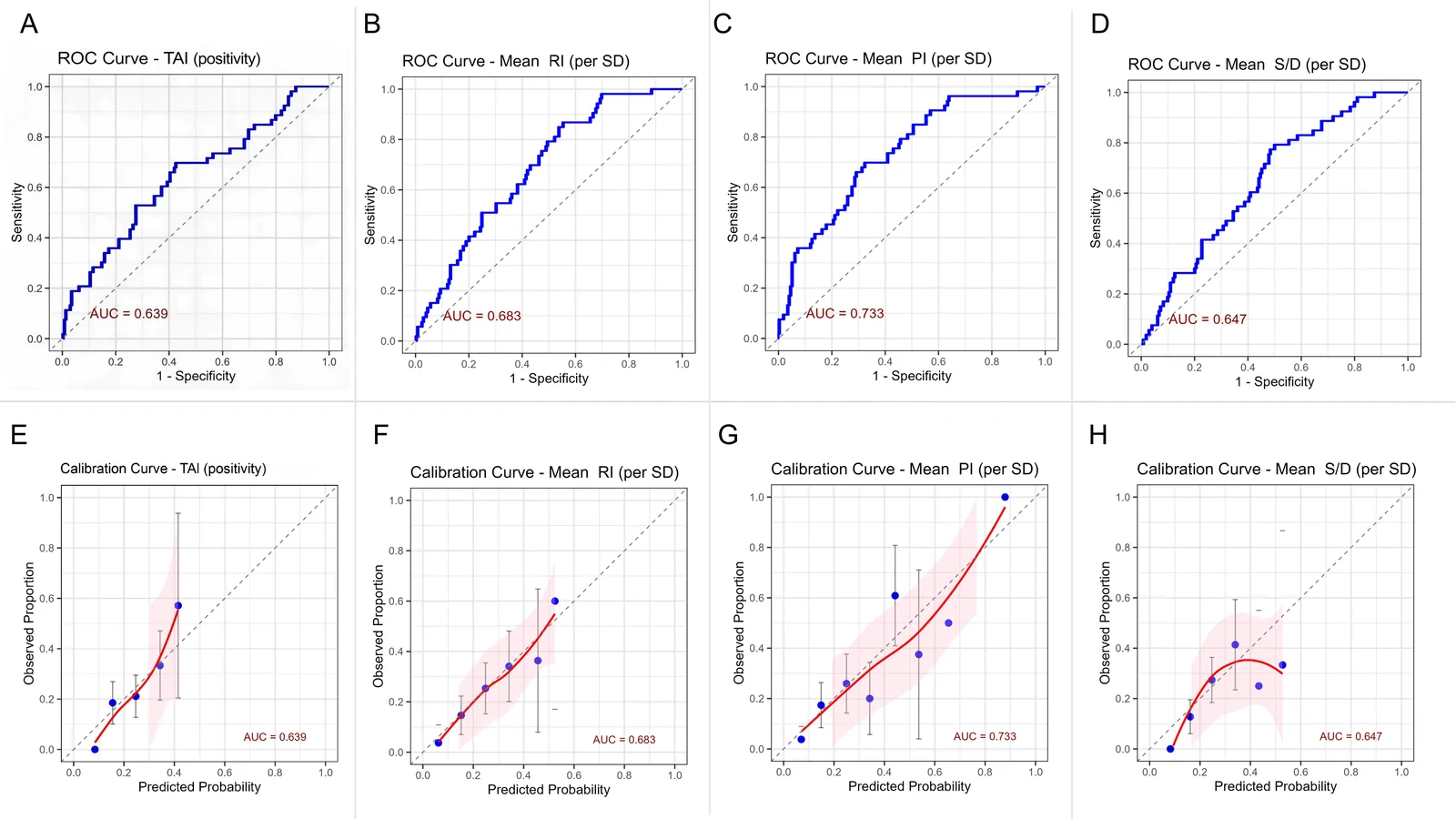
ROC curves and calibration curves of the models predicting subsequent pregnancy loss with TAI positivity, mean RI, mean PI and mean S/D. **(A–D)** ROC curves of TAI positivity, mean RI, mean PI and mean S/D. **(E–H)** Calibration curves of TAI positivity, mean RI, mean PI and mean S/D.

Multivariate logistic regression analysis (**Table 6**; **Figure 3**) revealed that TAI positivity (aOR=2.02, 95%CI 1.08-3.87, P = 0.028), mean RI (aOR=1.96, 95%CI 1.3-3.00, P <0.001), mean PI(aOR=2.24, 95%CI 1.58-3.30, P <0.001), and mean S/D (aOR=1.40, 95%CI 1.03-1.90, P = 0.032) were independent predictors of subsequent pregnancy loss after adjusting for maternal age, BMI, number of prior pregnancy losses, and TSH.

**Table 6:** Multivariate logistic regression analysis of the association between TAI, uterine artery Doppler parameters, and subsequent pregnancy loss.

Variable	OR	aOR	95% CI	*P* value
TAI (positivity)	**2.09**	**2.02**	**1.08-3.87**	**0.028**
Mean RI (per SD)	**2.00**	**1.96**	**1.30-3.00**	**<0.001**
Mean PI (per SD)	**2.19**	**2.24**	**1.58-3.30**	**<0.001**
Mean S/D (per SD)	**1.46**	**1.40**	**1.03-1.90**	**0.032**

Adjusted for maternal age, BMI, number of prior pregnancy losses, and TSH. OR and aOR for uterine artery parameters represent the change in risk per 1-SD increase. *P* values <0.05 were shown in bold. RI, resistance index; PI, pulsatility index; S/D, systolic/diastolic ratio; OR, odds ratio; aOR, adjusted OR.

## 4 Discussion

Recurrent pregnancy loss (RPL) affects 1-3% of women of childbearing age and poses a significant clinical challenge (2, 14). Thyroid autoimmunity (TAI) is frequently observed in women with recurrent pregnancy loss (RPL), but its relationship with uterine artery hemodynamics and subsequent pregnancy outcomes has not been directly investigated. The present study demonstrates that thyroid autoimmunity (TAI) is significantly associated with increased uterine artery blood flow resistance in non-pregnant women with unexplained recurrent pregnancy loss (URPL), and that both TAI positivity and elevated uterine artery Doppler parameters independently predict adverse subsequent pregnancy outcomes. Our study directly investigated the association between TAI and uterine artery hemodynamics in the non-pregnant state among URPL women, providing a potential mechanistic link between thyroid autoimmunity and reproductive failure. We are not aware of a previous study that has examined.

Our finding that TAI women had significantly higher uterine artery RI and PI values compared to non-TAI women extends previous observations regarding uterine artery hemodynamics in RPL populations. Yang et al. reported that impaired uterine artery blood perfusion may be an underlying pathology to RPL and can serve as an independent risk factor for pregnancy failure (5). Similarly, Pang et al. demonstrated that URPL women had significantly higher PI, RI, and S/D values compared to controls, with a PI cutoff of 2.6 and an RI cutoff of 0.86 effectively differentiating URPL from controls (6). Earlier studies also found elevated resistance indices in the mid-luteal phase of women with RPL, although they did not evaluate thyroid antibody status (15, 16). Our results corroborate these findings and further identify TAI as a potential contributor to elevated uterine artery resistance. Notably, TPOAb and TGAb titers showed weak positive correlations with uterine artery RI (r = 0.149 to 0.265), accounting for less than 6% of the variance in RI. These correlations are consistent with, but do not establish, a graded relationship.

The biological plausibility of this association is supported by emerging evidence on the effects of thyroid autoimmunity on placental and endometrial vascular biology. Spinillo et al. found that first-trimester TPOAb positivity was associated with increased rates of abnormal uterine artery Doppler PI and placental features of maternal vascular malperfusion (MVM), and this association was independent of TSH concentration (13). More recently, a study on placental angiogenic factors demonstrated that antithyroglobulin and antiperoxidase antibodies can negatively influence pregnancy outcomes by disturbing the placentation process and triggering an imbalance in angiogenic factors (17). These observations suggest that thyroid antibodies may exert direct effects on the vascular endothelium, potentially through immune-mediated endothelial dysfunction or altered angiogenesis. In the non-pregnant state, such vascular effects could manifest as increased basal resistance in the uterine arteries, as observed in our study.

The mechanisms by which TAI increases uterine artery resistance likely involve multiple interconnected pathways. First, thyroid autoantibodies may accompany a systemic low-grade inflammatory state, and chronic inflammatory can impair endothelial function and increase vascular tone, which would be expected to raise resistance indices (18–20). We did not measure inflammatory markers in this cohort, so this pathway remains hypothetical. Second, thyroid autoimmunity has been linked to endometrial immune dysregulation. Xie et al. demonstrated that autoimmune thyroid disease disrupts immune homeostasis in the endometrium during the window of implantation, with altered proportions of immune cell subsets and abnormal expression of chemokines (21). This immune imbalance may affect local vascular remodeling and endometrial receptivity. Third, shared biomarkers between Hashimoto’s thyroiditis and recurrent miscarriage, such as CFL1 and TRAPPC1, have been identified through transcriptomics analysis by Cheng et al. (22), suggesting common pathogenic pathways involving immune dysregulation and cell adhesion molecules. These molecular intersections may contribute to both thyroid autoimmunity and impaired uterine perfusion. Additionally, the role of antithyroid antibodies in activating complement and inducing endothelial injury has been discussed by Matalon et al. and reviewed in depth by Kyrilli et al. (7, 23).

Our follow-up data revealed that TAI women had significantly higher rates of subsequent pregnancy loss (28.7% vs. 16.0%) and lower live birth rates (71.3% vs. 84.0%) compared to TAI-negative women. These findings align with a recent meta-analysis by Quan et al., which demonstrated that TAI is associated with a higher risk of pregnancy loss in subsequent pregnancies among women with RPL (risk ratio: 1.46) (12). A 2024 meta-analysis further confirmed that women with anti-TPO and/or anti-TG antibodies have a higher risk of recurrent miscarriage than antibody-negative women (24). Our finding that TAI is independently associated with pregnancy loss (adjusted OR 2.02) is robustly corroborated by the independent validation from our previous non-overlapping cohort study (Reference 21: adjusted OR 2.953). The consistency of effect sizes across these two geographically and temporally distinct, non-overlapping cohorts provides compelling evidence for the generalizability of this association, while the novelty of the current report lies in the added predictive value of Doppler assessment (25). Earlier large-scale meta-analyses have already established TAI as a risk factor for miscarriage and adverse pregnancy outcomes, while prospective evidence from a well-characterized cohort has further corroborated this association (26–28). Our study extends these findings by showing that TAI status and pre-conception uterine artery Doppler indices are each associated with subsequent pregnancy loss after mutual adjustment. We did not perform a formal mediation analysis; therefore, the data do not support the inference that the effect of TAI on pregnancy outcome is mediated by uterine artery resistance. This hypothesis requires testing in an adequately powered study designed for that purpose.

The logistic regression analysis identified TAI positivity, mean RI, mean PI, and mean S/D as independent predictors of subsequent pregnancy loss after adjusting for age and BMI. When standardized per one standard deviation increase, the adjusted odds ratios were 1.96 (95% CI: 1.30-3.00) for mean RI, 2.24 (95% CI: 1.58-3.30) for mean PI and 1.40 (95% CI: 1.03-1.90) for mean S/D. These findings suggest that uterine artery Doppler parameters have significant predictive value for pregnancy outcomes in URPL patients, consistent with previous studies demonstrating that elevated uterine artery resistance indices are associated with increased risk of pregnancy failure (5, 6, 15, 16). The fact that both TAI status and Doppler parameters remained significant in the multivariate model suggests that TAI may influence pregnancy outcomes through both direct effects and indirect effects mediated by increased vascular resistance.

The clinical implications of our findings are substantial. First, uterine artery Doppler assessment could serve as a valuable tool for risk stratification in URPL patients with TAI. Women with elevated RI/PI values may benefit from closer monitoring or prophylactic interventions. Second, our results raise the question of whether therapies targeting uterine perfusion, such as low-dose aspirin, which has been shown to improve mid-luteal phase uterine artery blood flow in RPL patients (29), could be particularly beneficial in TAI women. Third, the identification of TAI as a modifiable risk factor for increased uterine artery resistance suggests that immunomodulatory approaches might have a role in improving pregnancy outcomes, although this requires further investigation. The meta-analysis of levothyroxine in euthyroid TPOAb-positive women has shown some benefit in reducing miscarriage (30), but data specific to URPL populations are still limited.

Several limitations of this study should be acknowledged. First, the retrospective design precludes establishing causality, and residual confounding cannot be excluded, and the frequency-matched enrollment design means that the reported clinical pregnancy, subsequent loss, and live birth rates are conditional on this sampling scheme and may not be generalizable as population estimates. Second, we did not measure additional markers of endothelial function or inflammation that could help elucidate the underlying mechanisms. Third, the study was conducted at a single center, and the generalizability of our findings to other populations requires validation. Forth, we did not assess partner semen parameters, coital frequency, or tubal patency, which may influence conception rates. In addition, we did not assess whether interventions targeting thyroid autoimmunity or uterine perfusion could modify the observed risks. Future prospective multicenter studies are warranted to evaluate whether interventions aimed at reducing uterine artery resistance or modulating thyroid autoimmunity can improve pregnancy outcomes in this high-risk population.

Despite these limitations, our study provides important insights into the relationship between thyroid autoimmunity and uterine hemodynamics in URPL. The finding that TAI is associated with increased uterine artery resistance in the non-pregnant state suggests that the vascular effects of thyroid autoimmunity may be established before conception, potentially predisposing to implantation failure and early pregnancy loss. This has implications for preconception counseling and management of URPL patients with TAI.

## 5 Conclusion

TAI positivity and elevated uterine artery Doppler indices (mean RI, PI and S/D) were each associated with subsequent pregnancy loss and a lower live birth rate, whereas the clinical pregnancy rate did not differ by antibody status. Uterine artery Doppler assessment may help stratify reproductive risk in TAI-positive URPL patients and guide potential therapeutic interventions.

## Data Availability

The original contributions presented in the study are included in the article/**Supplementary Material**. Further inquiries can be directed to the corresponding author.
