# Supplementary material for: Thyroid autoimmunity is associated with increased uterine artery blood flow resistance and predicts adverse pregnancy outcomes in women with unexplained recurrent pregnancy loss: a retrospective cohort study
Source: Front Endocrinol (Lausanne). 2026 Sep 7;17:1928429. doi: 10.3389/fendo.2026.1928429 (PMC13590354; doi:10.3389/fendo.2026.1928429)
Supplement: Supplementary file 1 [file Supplementaryfile1.doc]

### **Supplementary Tables and Figures**

### **Supplementary Tables S1 STROBE Checklist for this retrospective Cohort Studies**

| **Section/Topic** | **Item#** | **Recommendation** | **Reported**  **(Yes/No/NA)** | **Location in Manuscript** |
| --- | --- | --- | --- | --- |
| **Title and abstract** | 1 | (a) Indicate the study’s design with a commonly used term in the title or abstract | Yes | Title: “…retrospective cohort study”; Abstract: clearly states “retrospective cohort study”. |
|  |  | (b) Provide in the abstract an informative and balanced summary of what was done and what was found | Yes | Abstract includes background, objectives, methods (sample size, antibody definition, Doppler parameters), main results (RI/PI/S/D comparisons, pregnancy outcomes), and conclusion. |
| **Introduction** | 2 | Explain the scientific background and rationale for the investigation being reported | Yes | Introduction (paragraphs 1‑3) describes RPL, URPL, uterine artery resistance, TAI, and existing research gaps. |
|  | 3 | State specific objectives, including any prespecified hypotheses | Yes | Last paragraph of Introduction: “…aimed to explore the association between TAI and uterine artery Doppler parameters…” and to predict subsequent pregnancy outcomes. |
| **Methods** |  |  |  |  |
| **Study design** | 4 | Present key elements of study design early in the paper | Yes | Section 2.1 explicitly states “retrospective cohort study” and describes the enrolment period and setting. |
| **Setting** | 5 | Describe the setting, locations, and relevant dates | Yes | Section 2.1 gives hospital, department, enrolment period (June 2024 – October 2025) and follow‑up censoring date (June 30, 2026). |
| **Participants** | 6 | (a) Give the eligibility criteria, and the sources and methods of selection of participants | Yes | Section 2.1 defines URPL, lists exclusion criteria (anticoagulants, immunomodulators, hormones, abnormal thyroid function, diabetes, hypertension, ART, BMI≥28, current pregnancy). |
|  |  | (b) For cohort studies, describe the follow‑up method | Yes | Section 2.4 describes follow‑up (medical record review until clinical pregnancy, 12 months without conception, or census date). |
| **Variables** | 7 | Clearly define all outcomes, exposures, predictors, potential confounders, and effect modifiers | Yes | Exposure (TAI definition), outcomes (clinical pregnancy, subsequent loss, live birth), confounders (age, BMI, prior losses, TSH) are defined in Sections 2.2, 2.4, and 3.6. |
| **Data sources/ measurement** | 8 | For each variable of interest, give sources of data and details of measurement (e.g., laboratory, ultrasound), and if applicable, comparability of methods | Yes | Section 2.2 details laboratory assays (Roche e801, c702) and reference ranges; Section 2.3 details ultrasound equipment, probe, technique, and intra‑observer reproducibility (ICC). |
| **Bias** | 9 | Describe any efforts to address potential sources of bias | Yes | Sonographer was blinded to antibody results (Section 2.3); Firth penalised logistic regression and AIC‑based stepwise selection were used to reduce sparse‑data bias (Section 2.5). |
| **Study size** | 10 | Explain how the study size was arrived at | Yes | Section 2.5 states the sample was determined by number of eligible women enrolled, and provides power calculation (80% power to detect RI difference of 0.02). |
| **Quantitative variables** | 11 | Explain how quantitative variables were handled in the analyses (e.g., categorisation) | Yes | Continuous variables are presented as median (IQR); in regression, Doppler indices were per 1‑SD increase (Table 6); antibody positivity used a fixed cut‑off (60 IU/mL). |
| **Statistical methods** | 12 | (a) Describe all statistical methods, including those used to control for confounding | Yes | Section 2.5 describes normality tests, t‑tests/Wilcoxon, chi‑square, Spearman correlation, logistic regression (Firth penalty, AIC stepwise, forced adjustment), AUC, calibration, bootstrap internal validation. |
|  |  | (b) Describe any subgroup analyses or sensitivity analyses | No | Not explicitly reported. |
|  |  | (c) Explain how missing data were addressed | N/A | Complete data; no missingness mentioned. |
| **Results** |  |  |  |  |
| **Participants** | 13 | (a) Report numbers of individuals at each stage of study (e.g., screening, enrolment, follow‑up, analysis) | Yes | Section 3.1 gives 486 enrolled (242 TAI, 244 non‑TAI); Section 2.4 indicates 240 achieved clinical pregnancy. |
|  |  | (b) Give reasons for non‑participation or loss to follow‑up | N/A | No loss to follow‑up (all followed via medical records). |
|  |  | (c) Consider use of a flow diagram | No | Not provided, but textual description is clear. |
| **Descriptive data** | 14 | (a) Give characteristics of study participants (e.g., demographic, clinical) | Yes | Table 1 reports age, BMI, prior losses, thyroid function, metabolic parameters, and antibody levels. |
|  |  | (b) Indicate number of participants with missing data for each variable | N/A | Complete data. |
| **Outcome data** | 15 | Report numbers of outcome events or summary measures | Yes | Table 4 gives non‑pregnancy, subsequent loss, and live birth counts/rates; Table 5 gives Doppler indices by outcome. |
| **Main results** | 16 | (a) Give unadjusted and adjusted estimates and their precision (e.g., 95% CI), and specify which confounders were adjusted for | Yes | Table 6 provides OR and aOR with 95% CI; adjustments (age, BMI, prior losses, TSH) are listed in the footnote. |
|  |  | (b) If categorised, define the reference category | Yes | TAI reference = non‑TAI; continuous variables per 1‑SD increase. |
|  |  | (c) For analytical results, consider translating estimates of relative risk into absolute risk | No | Not performed. |
| **Other analyses** | 17 | Report other analyses done (e.g., subgroup, interaction, sensitivity) | No | Not reported. |
| **Discussion** |  |  |  |  |
| **Key results** | 18 | Summarise key results with reference to study objectives | Yes | First paragraph of Discussion summarises the main findings (TAI associated with higher resistance; both TAI and Doppler indices independently predict loss). |
| **Limitations** | 19 | Discuss limitations of the study, taking into account sources of potential bias or imprecision | Yes | Section 4 lists: retrospective design, lack of inflammatory markers, single‑centre, no male factor/tubal assessment, no intervention evaluation. |
| **Interpretation** | 20 | Give a cautious overall interpretation considering objectives, mechanisms, alternative explanations, and comparison with previous studies | Yes | Discussion provides in‑depth mechanistic interpretation (inflammation, immune dysregulation, vascular effects) and compares with prior literature (Yang, Pang, Spinillo, etc.). |
| **Generalisability** | 21 | Discuss the generalisability (external validity) of the study results | Yes | Mentions single‑centre limitation and suggests multi‑centre validation; however, consistency with a non‑overlapping cohort supports generalisability (Discussion, paragraphs 3 and 5). |
| **Other information** |  |  |  |  |
| **Funding** | 22 | Give the source of funding and the role of the funders for the present study | Yes | “Acknowledgments and Funding statement” specifies grant number (LHGJ20210595); role of funders not stated. |

**Supplementary Table S2 The log-rank test results of Kaplan-Meier analysis of time to clinical pregnancy.**

|  | **χ²** | ***df*** | ***P* value** |
| --- | --- | --- | --- |
| **Log Rank (Mantel-Cox)** | 6.805 | 1 | 0.009 |
| **Breslow (Generalized Wilcoxon)** | 11.668 | 1 | 0.001 |

**Supplementary Table S3 Comparison of pregnancy rate between TAI group and non-TAI group.**

| **Outcomes** | **TAI (n=242)** | **Non-TAI (n=244)** | **P value** |
| --- | --- | --- | --- |
| **Non-pregnancy rate** | 127(52.5%) | 119(48.8%) | 0.379 |
| **Clinical pregnancy rate** | 103 (42.6%) | 117(48.0%) |  |
| **biochemical pregnancy rate** | 12(5.0%) | 8(3.3%) |  |

Values are n/N (%). P values <0.05 were shown in bold.

**Supplementary Table S4 Multivariate logistic regression analysis of the association between TAI, uterine artery Doppler parameters, and subsequent pregnancy loss excluded pregnancy loss at than 12 gestational weeks.**

| **Variable** | **OR(95%CI)** | ***P*** | **aOR(95%CI)** | ***aP*** | **AUC** |
| --- | --- | --- | --- | --- | --- |
| **TAI (positivity)** | 2.07 (1.1-3.95) | **0.023** | 1.98 (1.04-3.85) | **0.037** | 0.6351 |
| **Mean RI (per SD)** | 2.06 (1.41-3.1) | **<0.001** | 2.01 (1.34-3.13) | **<0.001** | 0.6863 |
| **Mean PI (per SD)** | 2.12 (1.5-3.07) | **<0.001** | 2.16 (1.52-3.19) | **<0.001** | 0.7264 |
| **Mean S/D (per SD)** | 1.46 (1.09-1.97) | **0.011** | 1.39 (1.02-1.9) | **0.038** | 0.6471 |

Adjusted for maternal age, BMI, number of prior pregnancy losses, and TSH. OR and aOR for uterine artery parameters represent the change in risk per 1-SD increase. *P* values <0.05 were shown in bold. RI, resistance index; PI, pulsatility index; S/D, systolic/diastolic ratio; OR, odds ratio; aOR,adjusted OR. aP, adjusted *P* value.

**Supplementary Figures**

**Supplementary Figure S1 The participant flow diagram of this study. PL, pregnancy loss; LB, live birth.**

**Supplementary Figure S2 Kaplan-Meier curve of time to clinical pregnancy.**

**Supplementary Figure S3 The scatter plot of RI against S/D for the whole cohort.**
